# Face and Content Validity of Tympanostomy Tube Simulator With Variable Canal Size

**DOI:** 10.1002/oto2.70066

**Published:** 2025-01-05

**Authors:** Aurelia S. Monk, Landon Larabee, Daniel R. Bacon, Andrew Woodard, Adam J. Kimple, Christine E. DeMason

**Affiliations:** ^1^ Department of Otolaryngology–Head and Neck Surgery University of North Carolina Chapel Hill North Carolina USA; ^2^ Department of Surgery The Ohio State University College of Medicine Columbus Ohio USA; ^3^ Department of Radiology University of North Carolina Chapel Hill North Carolina USA

**Keywords:** myringotomy, PET, resident training, simulator

## Abstract

Simulators allow junior otolaryngology residents to practice the delicate procedure of pressure equalization tube (PET) insertion. However, most simulators lack the ability to mimic the differing anatomic complexities between patients, such as variable external auditory canal (EAC) size. We developed a novel low‐cost, medium‐fidelity 3‐dimensional‐printed PET simulator with different EAC sizes to better reflect procedure complexity. Additionally, a variety of materials were tested to mimic the elastic modulus of the tympanic membrane, with “Press'n Seal” cling film being chosen for its cost‐effectiveness and tactile similarity. Ten otolaryngologists performed PET insertion on both EAC sizes followed by a survey to assess face and content validity. Results indicated both high face and content validity, with most participants agreeing the simulator provided a realistic experience and would be useful for training. While our study has a small sample size, our PET simulator adds a unique and valuable addition to PET training.

Pressure equalization tube (PET) insertion is the most common pediatric otolaryngology procedure.^1^, ^2^, ^3^ As such, PET insertion is one of the earliest and most frequent procedures junior otolaryngology residents are exposed to during training.

PET insertion is a delicate procedure given the small area of the external auditory canal (EAC) and tympanic membrane (TM) involved, with ease of insertion depending on physician's skill and patient anatomy. EAC size and anatomy differ between patients, commonly due to age,^4^ but certain populations, such as those with Down syndrome, are more likely to exhibit EAC stenosis.^5^ Stenosis may present a challenge for PET insertion, especially for physicians early in their training.

Several PET simulators have been created to provide residents with a method of practice but most only provide 1 anatomic model.^6^, ^7^, ^8^ To address this limitation, we developed a novel low‐cost, medium‐fidelity 3‐dimensional (3D)‐printed PET simulator with interchangeable EAC parts. This study aims to assess the face and content validity of this novel PET simulator.

## Material and Methods

**Figure 1:**
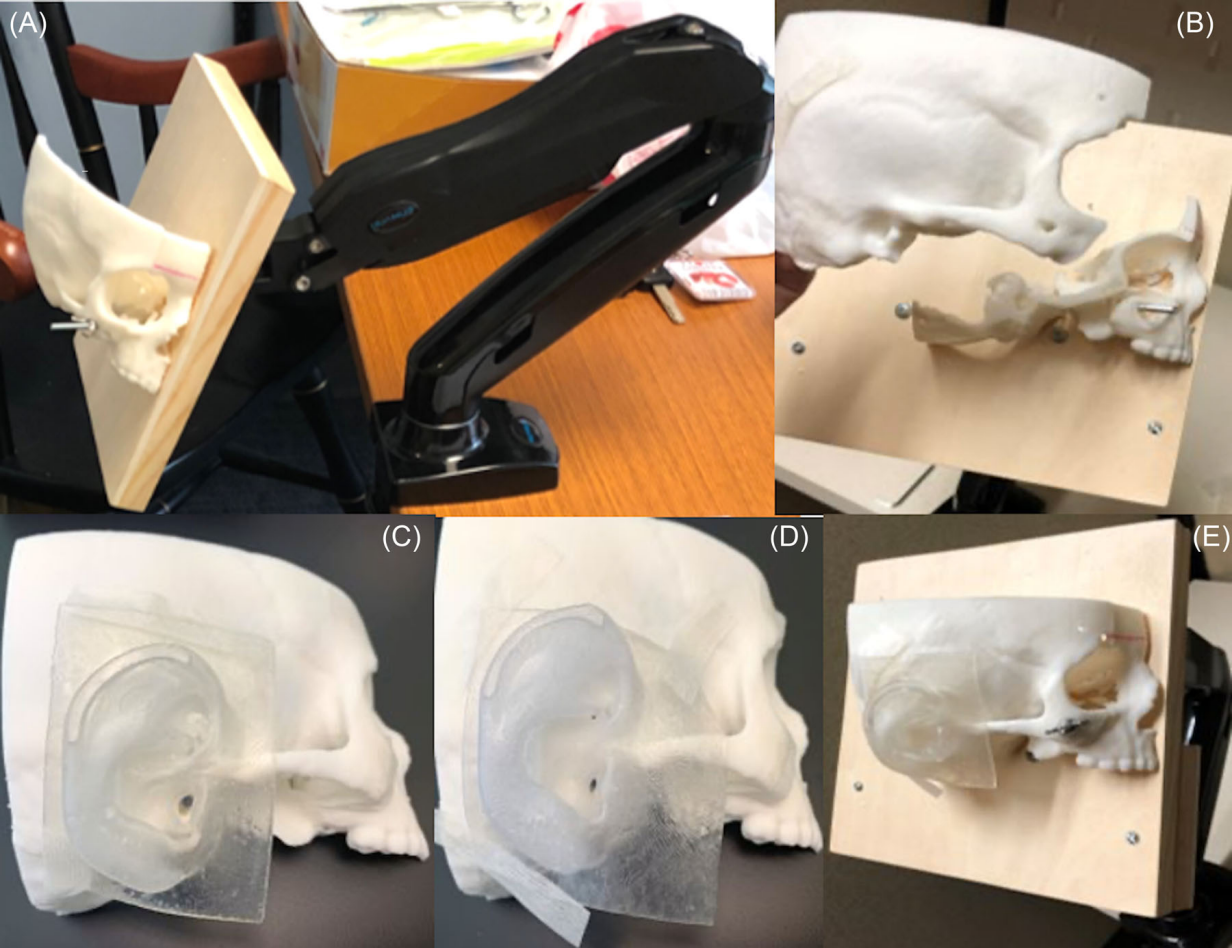
Photos of the model. (A) Simulator mounted on maneuverable computer stand. (B) Modular capability of the simulator allowing for change in ear models. (C) Normal EAC. (D) Narrow EAC. (E) Mounted simulator. EAC, external auditory canal.

A 3D‐printed PET simulator with 2 interchangeable EACs of different diameters was created, as seen in Figure 1C and D. The skull was 3D printed using a CT temporal bone of a 5‐year‐old and the EACs were digitally edited to create 2 models with different diameters. The normal EAC diameter was 5.6 mm. The alternative EAC had a diameter of 3.5 mm to mimic a narrow (<4 mm) EAC as in children with Down syndrome.^5^

The models were mounted to a computer monitor stand, as seen in Figure 1A. This allowed users to move the ear/head as in real life. Detailed instructions for building the simulator and additional photos are in the Supplemental Materials (Supplemental Figures S1‐S5, available online). Access to a handheld electric saw and drill are needed for construction. Table 1 lists the materials used to construct the simulator and their prices. The 3D printer, software, and personnel time were free at our institution. Excluding these elements, which are often available at other institutions for free or at a low cost and whose prices otherwise vary too significantly to estimate, the total cost of the simulator was $115.98.

**Table 1:** Cost breakdown of the materials used for the PET simulator

Item	Price, $ (USD)
Computer monitor stand	29.99
6 packs of wood canvas boards	14.99
Cornhole sandbags (to add weight to the computer monitor for controlled movement)	12.78
Gorilla wood glue	5.97
Press'n seal cling wrap	2.98
2 of 5/16th short screws	1.62
1 of 5/16th long screw	0.81
2 winged nuts	0.98
Steristrips	5.86
3D printing materials	40

The total cost was $115.98 without tax. The 3D‐printed skull was provided at no charge and access to power tools is needed.

Abbreviations: 3D, 3‐dimensional; PET, pressure equalization tube.

To complete the simulator, the “Press'n Seal” cling wrap was chosen to act as the TM. Initially, different materials were assessed to replicate the elastic modulus of the TM, including parafilm, pig intestine, latex gloves, Tegaderm, wafer paper, and “Press'n Seal” cling wrap. Though unable to replicate its exact elastic modulus, we selected “Press'n Seal” due to its accessibility, cost‐effectiveness, and tactile similarity to a TM. Small pieces of “Press'n Seal” were cut and placed over the middle ear space to represent the TM when using the simulator. A view through a microscope visualizing the “Press'n Seal” TM with successful tube insertion can be found in Supplemental Figure S6, available online.

Participants with experience in PET insertions were recruited to complete PET insertion on both EACs of the simulator. Four short videos demonstrating simulator setup, temporal bone switching, and PET insertion are included online as supplementary materials (Supplemental Videos [Link], [Link], [Link], [Link], available online). A postsimulation questionnaire assessing face and content validity based on a prior study was completed by each participant.^6^ Participants were asked the extent to which they thought each simulator component was an accurate representation of its real‐life counterpart, and if such components would be beneficial for training. The rating scale included: strongly agree, disagree, neither agree or disagree, agree, and strongly agree.

Face validity questions assessed the visual appearance of the different parts of the EAC and TM, placement of speculum, tactile feel of eardrum, and tube placement. Content validity questions assessed the speculum placement, microscope positioning, EAC and TM anatomy, myringotomy creation, and difficulty of procedure. This study was deemed to be Institutional Review Board (IRB)‐exempt by the University of North Carolina IRB.

## Results

**Figure 2:**
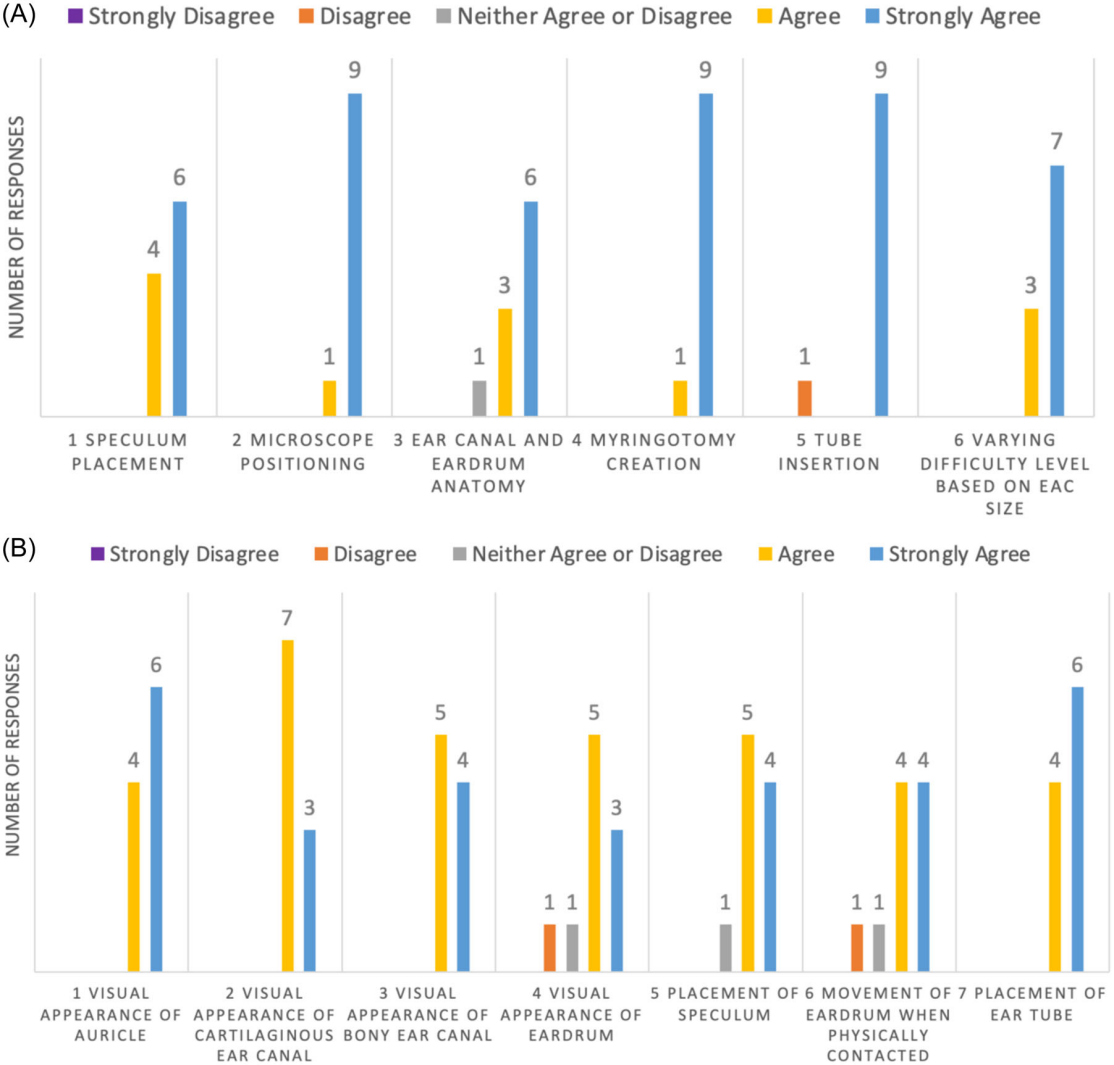
The majority of participants agreed that the simulator appropriately mimics the true surgical procedure for myringotomy with PET placement and would be beneficial for resident training. (A) Content validity. (B) Face validity. PET, pressure equalization tube.

Ten otolaryngologists—6 attending physicians, 2 fellows, and 2 upper‐level residents (≥fourth year of training)—were recruited. Results of the face validity questionnaire assessing if the simulator provided a realistic comparison to the actual procedure were as documented in Figure 2. All participants agreed the appearance of the auricle, cartilaginous ear canal, and bony ear canal, as well as the placement of the ear tube, were realistic. Eight of 10 agreed the appearance and movement of the eardrum were realistic when surgically manipulated. Nine of 10 agreed the placement of the speculum mimicked the real procedure.

Results of the content validity questionnaire assessing if the simulator provided useful teaching for residents were as follows: All participants agreed about the aspects of speculum placement, microscope positioning, myringotomy creation, and difficulty level based on EAC size. Nine of 10 participants agreed with the aspects EAC and TM anatomy and tube insertion (Figure 2).

## Discussion

Questionnaire responses were predominantly positive and suggested appropriate face and content validity for the PET simulator.

As discussed in the methods, we assessed several different materials in an attempt to best reflect the elastic properties of the TM while still taking into account accessibility and cost. The decision to use the “Press'n Seal” cling wrap was based mostly on its low cost and easy accessibility. Though our simulator did not score as high on factors related to the movement and visualization of the TM compared to other aspects, the majority (80%) still agreed that the “Press'n Seal” TM was realistic and beneficial for teaching. It is possible that other materials have closer elastic properties to a real TM but this may lead to increased cost of the simulator. Other studies have also had difficulty mimicking the properties of a TM.^6^, ^7^, ^8^ Of course, training programs can tailor their simulators based on preference using our model as a basis.

To our knowledge, this model is the first to provide options for differing EAC sizes thus allowing variable complexity in training. Additionally, the use of a maneuverable computer stand for mounting allows realistic positioning of the head and microscope.

One limitation of our study was the small sample size tested, though consistent with other PET simulators.^6^, ^7^, ^8^ Planned next steps will involve a skills assessment among novice operators using automated performance metrics.

## Conclusion

Simulation can play an essential role in preparing novice residents for PET. Our PET simulator adds a new level of complexity important in physician training. Though our study is small, it demonstrated our PET simulator of varying EAC sizes shows high face and content validity with the potential to provide a unique and valuable addition to PET training. We are happy to help any institution that wishes to build this simulator for their use.

## Author Contributions

**Aurelia S. Monk**, project design, experiment execution, data analysis, manuscript authoring and review; **Landon Larabee**, idea conception, project design, experiment execution, data analysis, manuscript authoring, and review; **Daniel R. Bacon**, idea conception, project design, experiment execution, data analysis, manuscript authoring, and review; **Adam J. Kimple**, idea conception, project design, experiment execution, manuscript authoring, and review; **Christine E. DeMason**, idea conception, project design, experiment execution, manuscript authoring, and review.

## Disclosures

### Competing interests

None.

### Funding source

None.

## Supporting information

Supporting information.

Supporting information.

Supporting information.

Supporting information.

Supporting information.
