# Supplementary material for: Face and Content Validity of Tympanostomy Tube Simulator With Variable Canal Size
Source: OTO Open. 2025 Jan 5;9(1):e70066. doi: 10.1002/oto2.70066 (PMC11764988; doi:10.1002/oto2.70066)
Supplement: Supplementary file 1 — Supporting information. [file OTO2-9-e70066-s004.docx]

**Supplemental Material**

*Simulator Construction Instructions*

*Access to a handheld electric saw and drill are needed to construct the simulator.

1. Gather your materials as listed in Table 1.
2. Open your monitor stand and set aside the included bolts and wrench used for attaching a monitor.
3. Take the movable portion of the monitor stand (where the monitor is designed to attach) and place it on the backside of a 10x10-inch wood canvas board. Center the monitor stand and trace the bolt holes in the monitor stand onto the wood canvas board.
4. Drill holes corresponding to the traced bolt holes into the wood canvas board. These will allow you to mount the simulator at the end.
5. Place the 3D-printed medial half skull so the most anterior part of the dentition is about 0.25cm from the edge of the 10x10-inch wood canvas board along the X-axis. Ensure it is centered along the y-axis.
6. Drill through the maxillary sinus using a long 5/16^th^-inch screw at a 45-degree angle inward (toward the inside of the skull). Ensure the screw penetrates the wood canvas board.
7. Insert a 5/16^th^-inch bolt from the back of the wood canvas board through the maxillary sinus.
8. Secure a winged nut onto the end of the bolt protruding through the sinus. Tighten until the 3D-printed skull is firmly compressed against the board. Placing the screw through the maxillary sinus allows the lateral component of the skull containing the EAC to be easily removed and exchanged by users.
9. Cut a separate 10x10-inch wood canvas board in half with an electric saw.
10. Attach the back portion of the cut wood canvas board to the back of the board containing the skull at the bottom edge (bottom to bottom) using the drill and two short 5/16^th^-inch screws. This creates a pocket for the sandbags.
11. Place the monitor stand against the holes traced earlier and insert the bolts, securing them with the corresponding nuts.
12. Place the monitor stand with the attached skull on a flat surface, like a table. Clamp to the table with the provided monitor attachments.
13. Place three sandbags (corn hole bags) in the pocket created earlier. The weight will balance the simulator by counteracting the spring-loaded mechanism of the monitor.
14. Swivel or remove the lateral component of the skull and add a small piece of “Press’n Seal over the middle ear space.
15. Re-attach or tighten the lateral component of the skull and proceed with PET insertion practice.

*For added support between the medial and lateral skull pieces, additional holes can be drilled in the posterior inferior aspect (mastoid tip area) of all skull pieces for zip tie placement. However, this prevents swiveling of the lateral temporal bone.

**Supplemental Figure 1.**


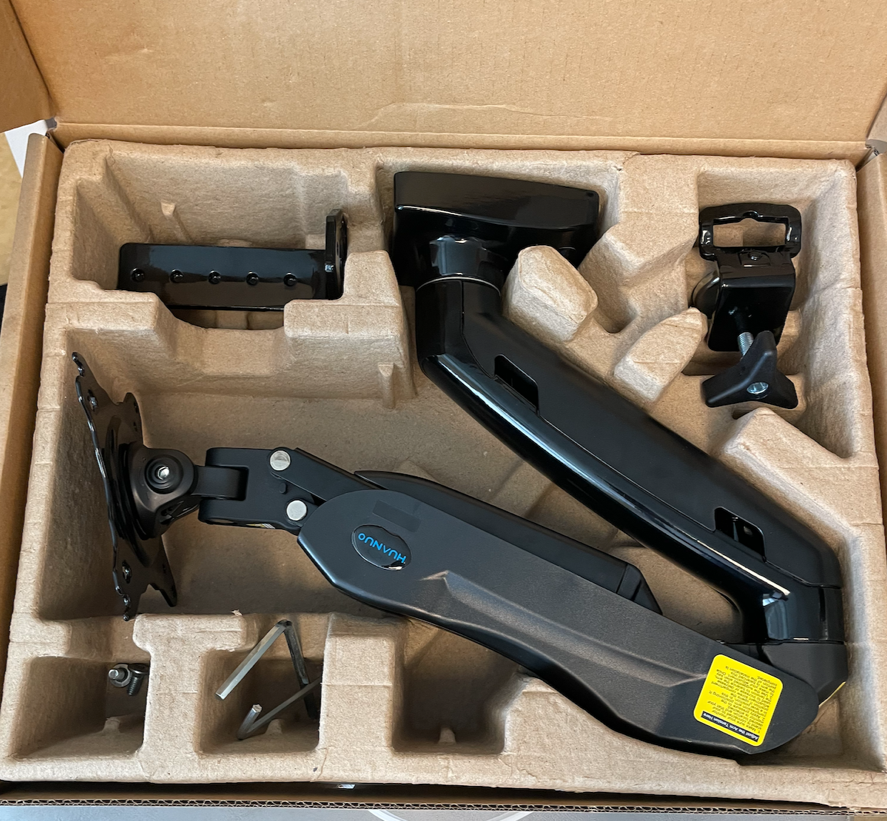


**Supplemental Figure 2.**


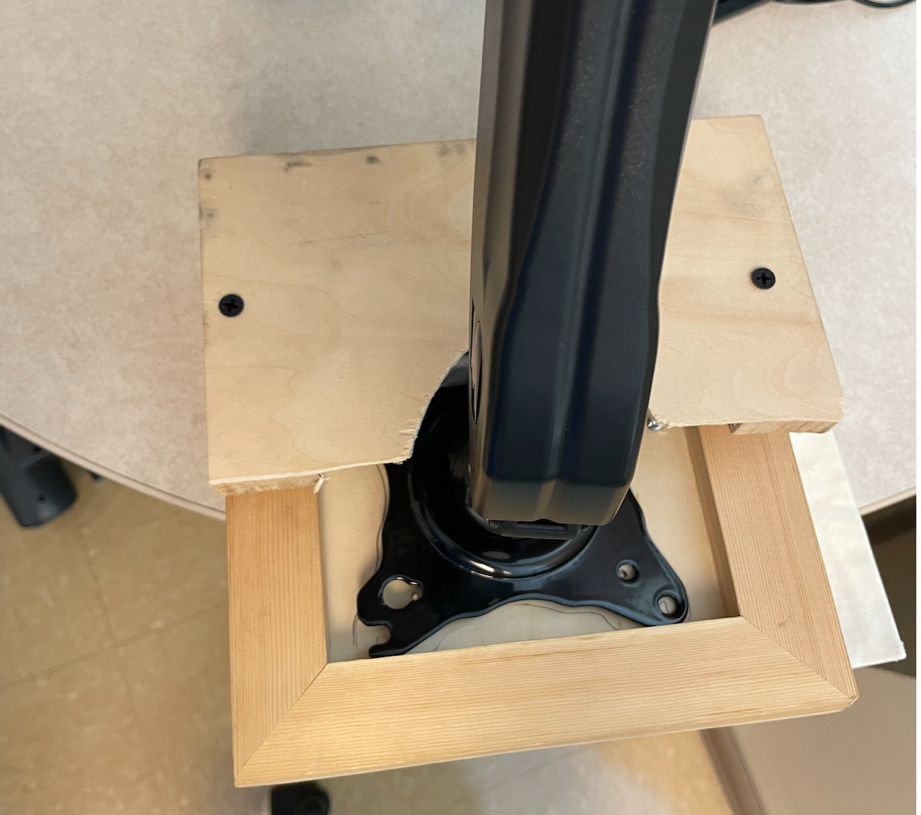


**Supplemental Figure 3.**

**
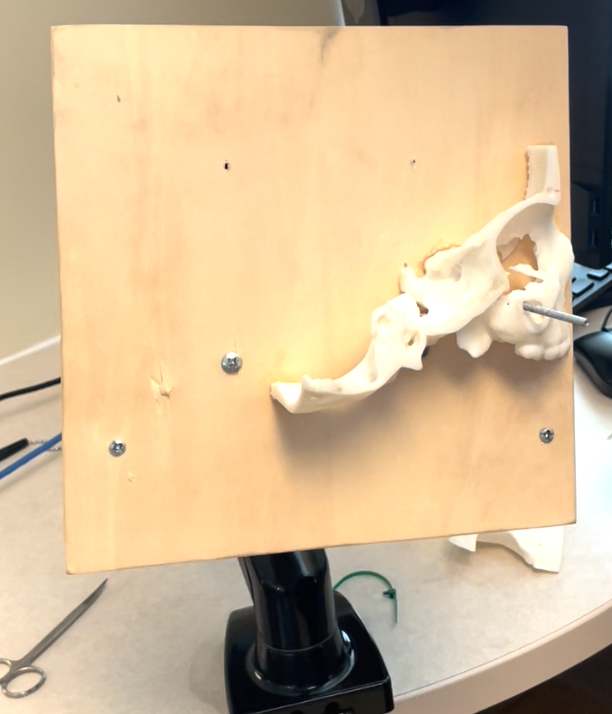
**
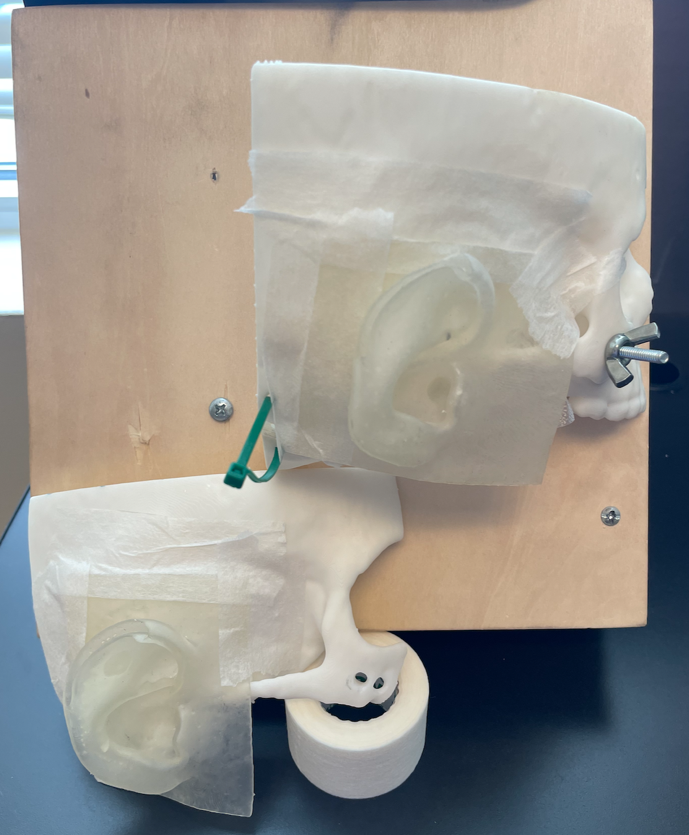


**B**

**A**

**Supplemental Figure 4.**


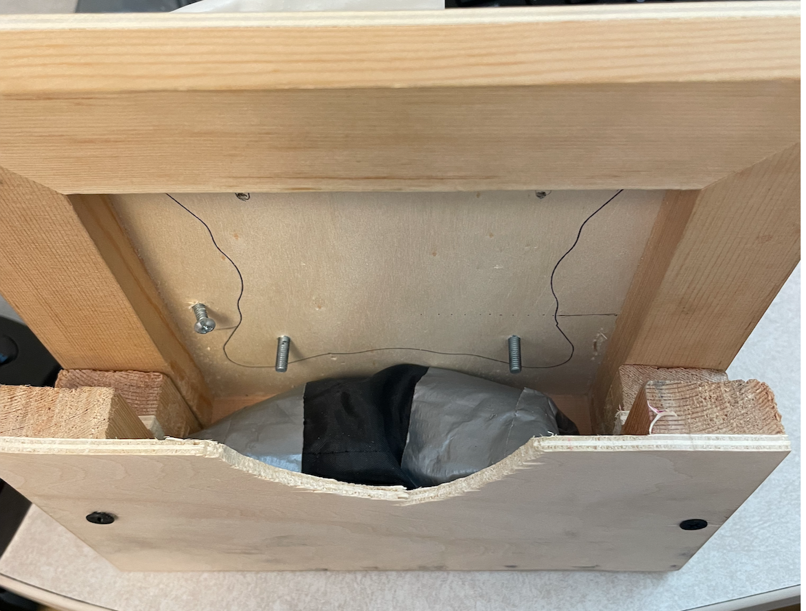

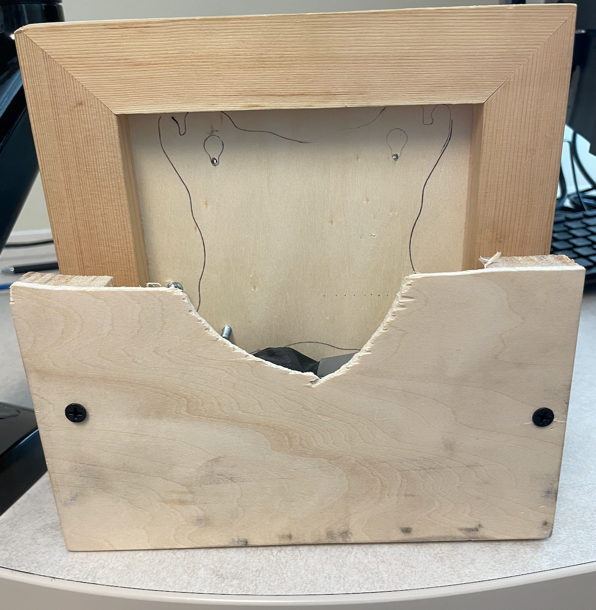


**A**

**B**

**
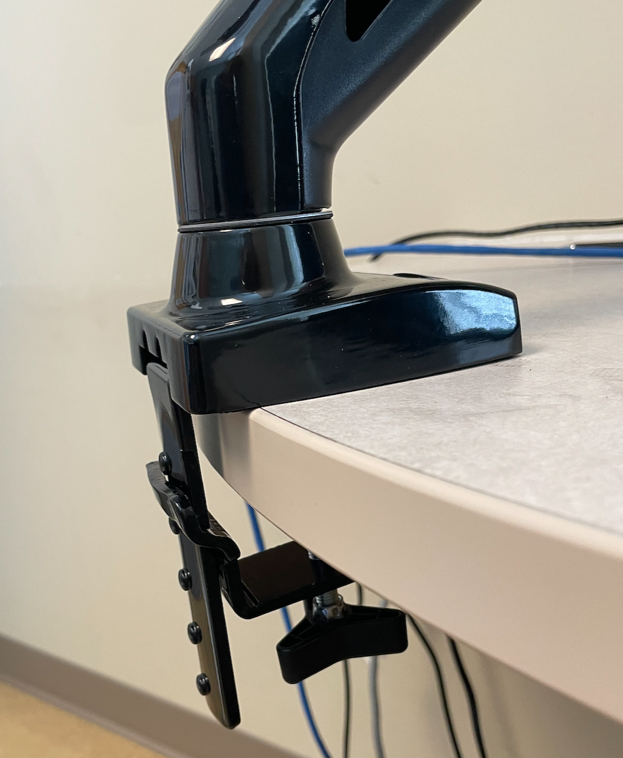
Supplemental Figure 5**.

**A**

**B**


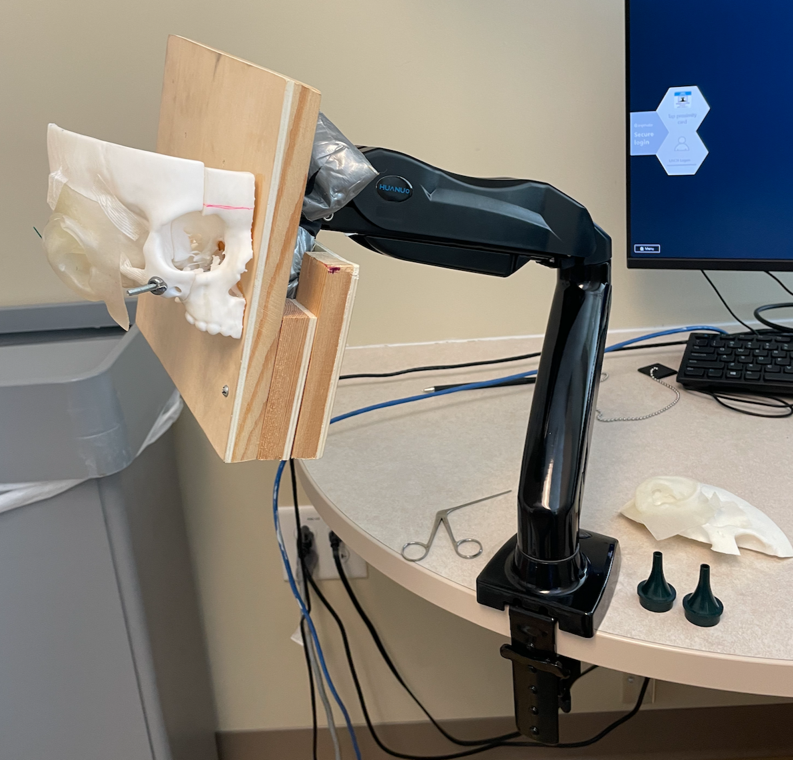


**Supplemental Figure 6.**

**
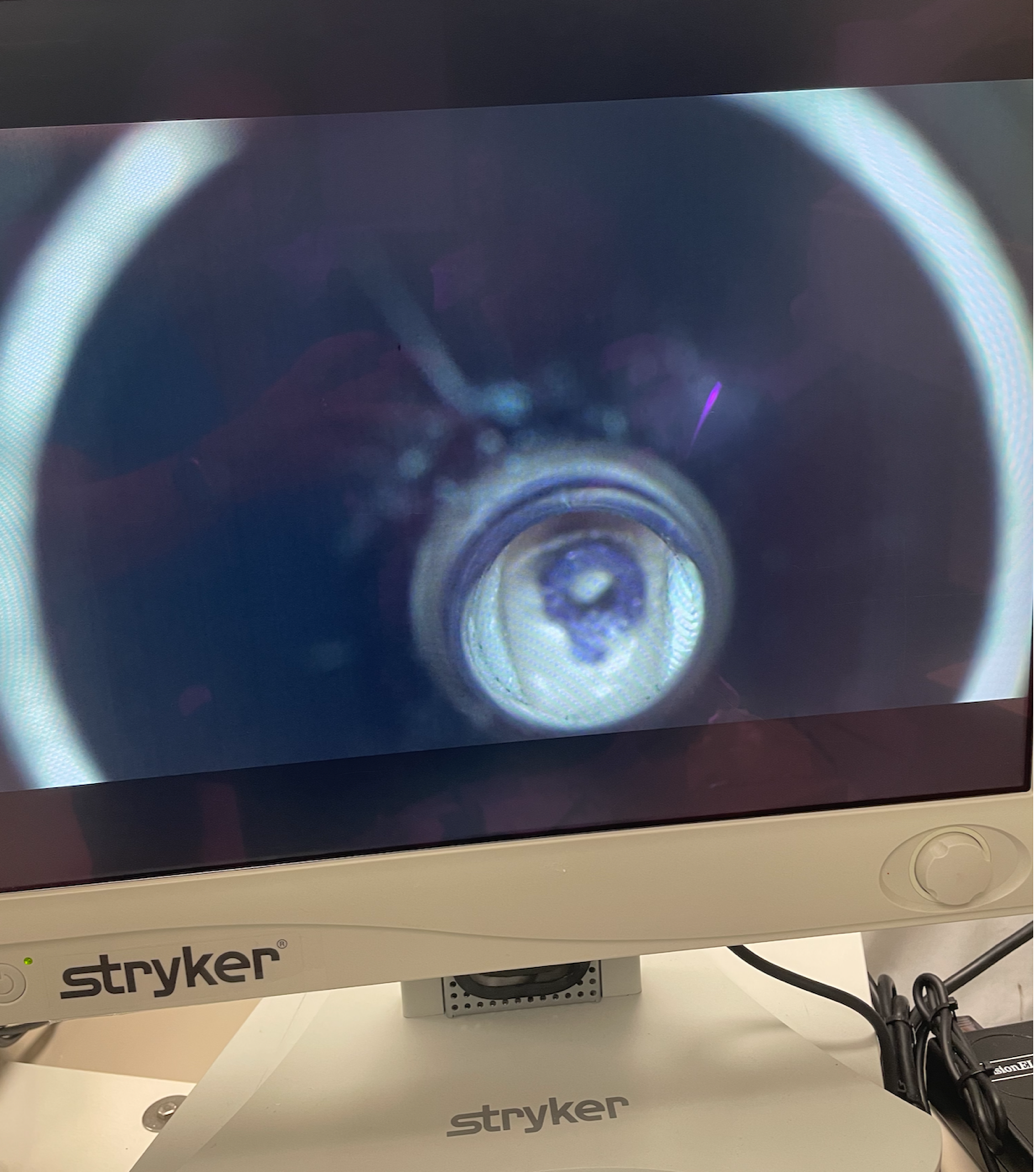
**
